# Black blood late gadolinium enhancement using combined T_2_ magnetization preparation and inversion recovery

**DOI:** 10.1186/1532-429X-17-S1-O14

**Published:** 2015-02-03

**Authors:** Tamer Basha, Sébastien Roujol, Kraig V Kissinger, Beth Goddu, Warren J Manning, Reza Nezafat

**Affiliations:** 1 Cardiology, BIDMC, Boston, MA, USA; 2 Harvard Medical School, Boston, MA, USA

## Background

Late gadolinium enhancement (LGE) imaging allows assessment of focal scar [1]. An inversion recovery based sequence is commonly used to achieve suppression of healthy myocardium signal. However, the blood pool and subendocardial scar typically have similar signal, making it difficult to distinguish subendocardial scar. Several methods have been proposed to increase the blood-scar contrast including a double inversion technique [2], and T_2_-prepared (T_2_prep) sequences [3,4]. However, all these approaches suffer from either reduced SNR or reduced scar-myocardium contrast. In this work, we propose a novel pulse sequence that uses an optimized combination of an inversion pulse and a T_2_prep composite pulse to simultaneously null both the healthy myocardium and blood signals, producing a black-blood LGE (BB-LGE) image without losing significant scar-myocardium contrast. We also developed a quick navigation sequence, analogous to the Look-Locker, to help determine the ideal nulling time before imaging.

## Methods

**Figure 1:**
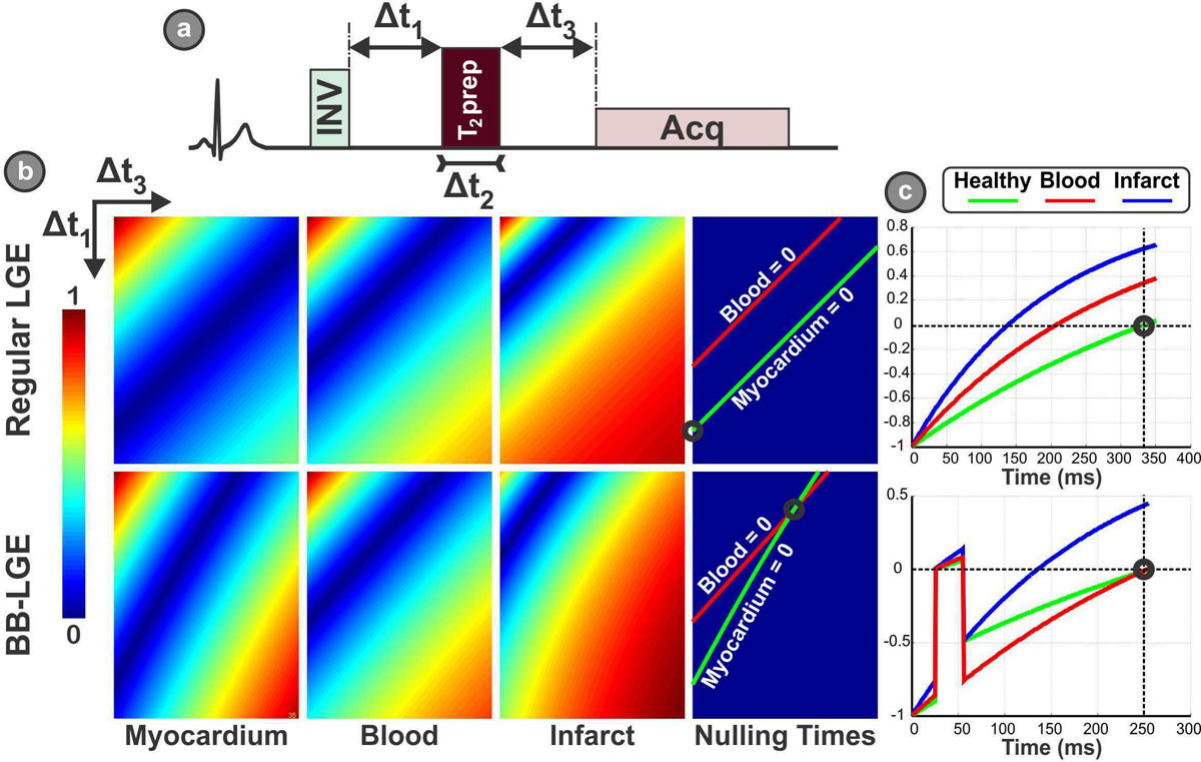
a) Schematic of the proposed pulse sequence, where a T_2_prep composite pulse is inserted between the inversion pulse and the data acquisition. Through changing the time of the T_2_prep composite (Δt_2_), the inversion-composite interval (Δt_1_) and composite-acquisition interval, the contrast between, healthy myocardium, blood pool and scar can be controlled. b) Simulated contrast maps that shows the contrast of the three tissues for different combinations of Δt_1_ and Δt_3_, in both standard LGE (Δt_2_=0), and the proposed BB-LGE (Δt_2_ =35ms). In conventional LGE, the null lines of myocardium and blood do not intersect at a common point, preventing simultaneously nulling of both tissues. However, in the BB-LGE, a common null point can be obtained. c) The signal evolution curve for the three tissues when using conventional LGE and the proposed sequence.

Figure 1.a shows a schematic of the proposed sequence. A T_2_prep pulse is inserted between the inversion pulse and the acquisition. Numerical simulation was conducted to simulate the effect of this pulse configuration on the signal intensity of healthy myocardium, blood, and infarcted myocardium assuming their T_1_ to be 500, 350, and 200 ms respectively. Six healthy adult subjects and 2 patients with suspected scar (35±21 y, 4 males) were imaged using a 1.5T Philips scanner. A standard LGE sequence was used to acquire a stack of five 2D short-axis slices, and one long-axis slice 20 min after Gd-contrast injection with the following parameters: FOV = 320×320mm^2^, slice gap = 5mm, in-plane resolution = 1.5×1.5 mm^2^, slice thickness = 10mm, TR/TE = 6.2/3.0ms, α = 25°, SENSE rate = 2, acquisition window = 80 ms, NSA = 2, breath-hold = 8s per slice. Then, the proposed BB-LGE, with the same sequence parameters, was used to acquire the same slices while setting the sequence timing to the null point of both the normal myocardium and the blood pool. The BB-LGE was preceded by a short navigation scan to determine the timing parameters that achieve the perfect nulling of both blood and myocardium signals.

## Results

**Figure 2:**
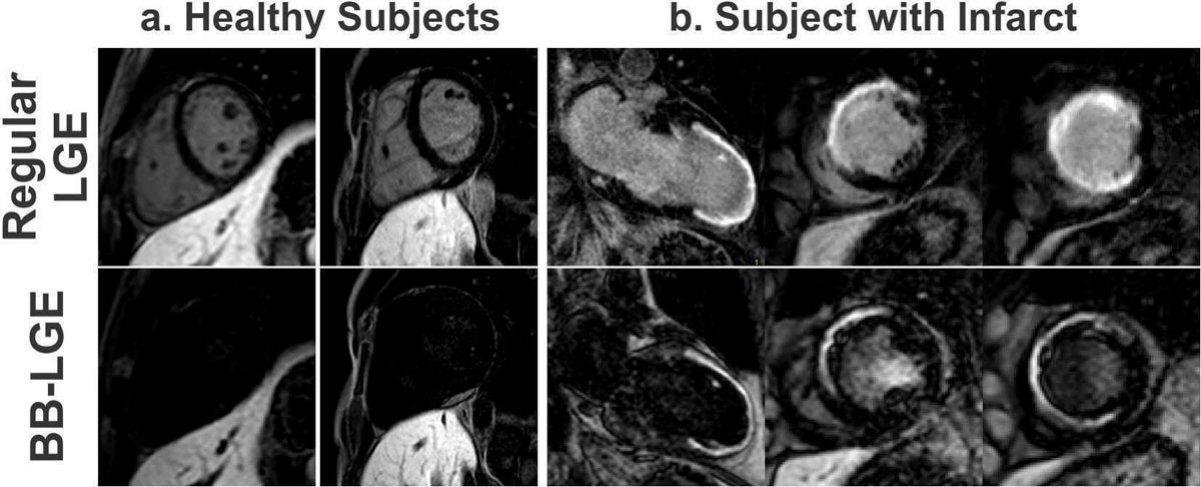
In-vivo results for the proposed BB-LGE compared with a conventional LGE sequence in the same subjects and slices. a) Two healthy subjects, where the proposed sequence successfully nulled both the myocardium and the blood pool. b) Example short-axis and long-axis slices from one infarct patient demonstrating suppression of LV blood signal with retention of the infarct enhancement, which facilitate the delineation of the scar territory, when compared to regular LGE.

Figure 1b shows the numerical simulation for signal intensity in healthy myocardium, blood and infarct tissues using both regular LGE sequence, and the proposed BB-LGE for Δt_2_=35ms. In the standard LGE sequence, there is no intersection that achieves a common null point for both myocardium and blood pool, while this null point can easily be obtained with the BB-LGE sequence. Figure 2 shows for the BB-LGE images examples in two healthy subjects (i.e. no infarct), and one patient with an infarction.

## Conclusions

A new BB-LGE sequence was developed to simultaneously null both healthy myocardium and blood pool in LGE sequences based on the higher T_2_ value of the blood.

## Funding

N/A.

## References

[B1] Kwong Circulation 2006 10.1161/CIRCULATIONAHA.105.609263 16505189

[B2] Foo MRM 2005

[B3] Kellman JMRI 2005

[B4] Liu JMRI 2008

